# Green‐up selection by red deer in heterogeneous, human‐dominated landscapes of Central Europe

**DOI:** 10.1002/ece3.9048

**Published:** 2022-07-04

**Authors:** Benjamin Sigrist, Claudio Signer, Sascha D. Wellig, Arpat Ozgul, Flurin Filli, Hannes Jenny, Dominik Thiel, Sven Wirthner, Roland F. Graf

**Affiliations:** ^1^ Wildlife Management Unit Institute of Natural Resource Sciences, ZHAW Zurich University of Applied Sciences Wädenswil Switzerland; ^2^ Population Ecology Research Group Department of Evolutionary Biology and Environmental Studies, University of Zurich Zurich Switzerland; ^3^ Valais Hunting, Fisheries and Wildlife Department Sion Switzerland; ^4^ Swiss National Park Zernez Switzerland; ^5^ Grisons Game and Fisheries Department Chur Switzerland; ^6^ St. Gall Conservation Hunting and Fisheries Department St. Gallen Switzerland

**Keywords:** green wave hypothesis, human disturbance, migration, net squared displacement, normalized difference vegetation index NDVI, remote sensing, sentinel 2, Switzerland

## Abstract

The forage maturation hypothesis (FMH) assumes that herbivores cope with the trade‐off between digestibility and biomass in forage by selecting vegetation at intermediate growth. The green wave hypothesis (GWH) extends the FMH to suggest how spatiotemporal heterogeneity in plant quality shapes migratory movements of herbivores. Growing empirical support for these hypotheses mainly comes from studies in vast landscapes with large‐scale habitat heterogeneity. It is unclear, however, to what extent ungulates surf green waves in human‐altered landscapes with small‐scale heterogeneity in terms of land use and topography. We used plant phenological proxies derived from Sentinel 2 satellite data to analyze the habitat selection of 93 collared red deer (*Cervus elaphus*) in montane and alpine habitats. Using a step selection analysis, we investigated how plant phenology, that is, the instantaneous rate of green‐up (IRG) and normalized difference vegetation index (NDVI), and a set of variables describing topography and human presence influenced red deer resource selection in open habitats. We learned that red deer selected areas with high biomass at green‐up and avoided habitats with possible exposure to human activity. Additionally, landscape structure and topography strongly influenced spatial behavior of red deer. We further compared cumulative access to high‐quality forage across migrant strategies and found migrants gained better access than residents. Many migratory individuals surfed the green wave, and their surfing behavior, however, became less pronounced with decreasing distance to settlements. Within the constraints of topography and human land use, red deer track spring green‐up on a fine spatiotemporal scale and follow the green wave across landscapes in migration movements. Thus, they benefit from high‐quality forage even in human‐dominated landscapes with small‐scale heterogeneity and vegetation emerging in a heterogenic, dynamic mosaic.

## Graphical abstract

In the human‐dominated landscapes of Central Europe, red deer surf the wave of spring green‐up, however, its habitat selection is strongly governed by complex topography, human presence and land use. About two thirds of the population under study exhibited migratory behaviour, thereby gaining better access to high quality forage than residential individuals.

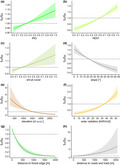

## 1 INTRODUCTION

In temperate environments with pronounced seasonality and fluctuating resources, access to high‐quality forage has proven to be a crucial factor for ungulate survival (Hurley et al., 2014), condition (Albon & Langvatn, 1992; Middleton et al., 2018), and reproduction (Middleton et al., 2018). Additionally, the distribution of high‐quality forage is an important driver of animal movement within and between seasonal ranges (Albon & Langvatn, 1992; Hebblewhite et al., 2008). The forage maturation hypothesis (FMH, Fryxell, 1991) is well established among concepts that drive migration, as it predicts that herbivores will select intermediate forage biomass (hereafter green‐up) to balance the trade‐off between forage quantity and quality (Hebblewhite et al., 2008). Climatic gradients cause spring green‐up to occur earlier at low elevations (or latitudes) and move as a “green wave” along these gradients, before later arriving at higher elevations (or latitudes). Hence, this green wave is believed to be a trigger for migration in species that are adapted to dealing with spatiotemporal variations in resource availability (Aikens et al., 2017).

The green wave hypothesis (GWH, Drent et al., 1978) predicts that migrating herbivores will track the leading edge of the spring green wave (termed “surfing the green wave”, Van Der Graaf et al., 2006) and has recently gained considerable attention in ungulate research. Some studies found evidence for ungulates surfing the green wave (Aikens et al., 2017; Merkle et al., 2016). However, ungulates from another study instead jumped the green wave, by moving quickly from their winter to their summer ranges (Bischof et al., 2012). As a result, they did not prolong the green wave exposure by surfing it, but by benefitting from spring green‐up at their summer ranges (Bischof et al., 2012). This behavior might be governed by constraints such as predation risk (Rivrud et al., 2018) or limited access to resources (Bischof et al., 2012) along the migratory route. It is currently unclear whether surfing or jumping is more beneficial and this will likely depend on various factors, such as landscape characteristics (Mysterud et al., 2017), reproduction status (Bischof et al., 2012), and social learning (Jesmer et al., 2018). However, there seems to be a consensus in the vast majority of the studies that migrating individuals outcompete resident in terms of access to high‐quality forage (Bischof et al., 2012; Mysterud et al., 2017).

**FIGURE 1:**
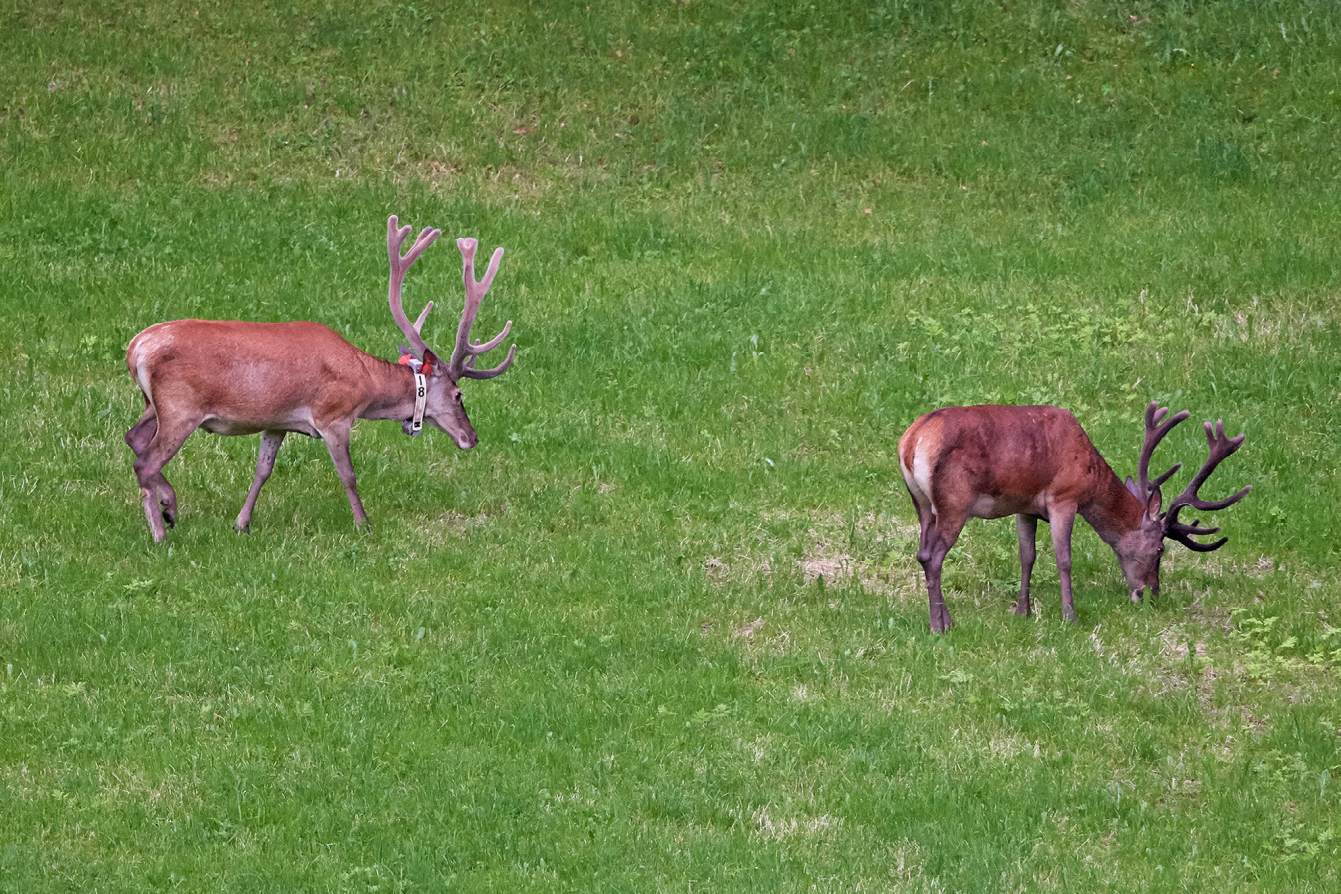
A collared male red deer (*Cervus elaphus*) grazes with a conspecific in a meadow in one of the study areas. Author: Markus P. Stähli

Most of the studies conducted to date have been located in vast, relatively sparsely populated areas, such as the Norwegian countryside (Bischof et al., 2012; Mysterud et al., 2017; Wildlife Conservation Society, 2005, population density: human footprint index = 6.8), the greater Yellowstone ecosystem and more broadly across the intermountain west, USA (Aikens et al., 2017; Merkle et al., 2016; Middleton et al., 2018; Wildlife Conservation Society, 2005, population density: human footprint index = 3.1). However, the influence of spring green‐up on ungulate habitat selection is unclear in landscapes where a small‐scale mosaic of complex topography, human land use, and predators govern the availability and accessibility of suitable foraging patches. In such landscapes, the green wave may not just move across the landscape in one direction but will rather emerge as a heterogenic, dynamic mosaic of patches shaped by topography (slope, aspect, and altitude) and varying intensities of land use. Furthermore, in populous areas, human activities may have a significant influence on spatiotemporal resource selection and migrant strategies of ungulates. Therefore, a better understanding is needed of how these factors constrain resource selection and animal movement within the GWH framework. In Central Europe, green wave tracking by red deer (*Cervus elaphus*; Figure 1) in the 2010s was unlikely to be constrained by predation risk, due to relatively low densities of wolf (*Canis lupus*) (Chapron et al., 2014). However, human disturbance can be seen as a form of predation risk (sensu: Frid & Dill, 2002), and therefore it is likely that certain factors, such as recreational activities and human infrastructure, have a constrictive effect. Various studies have shown red deer and elk (*Cervus canadensis*) avoid roads and trails (Coppes et al., 2017; Roberts et al., 2017) or alter their foraging behavior as a function of distance to human infrastructure (Ciuti et al., 2012). In some management systems, winter enclosures (Rivrud et al., 2016) and supplementary winter feeding (Coppes et al., 2017) may further influence space use. Additionally, man‐made alterations to foraging grounds, such as fertilization or mowing of meadows, may have considerable consequences on forage availability for red deer (Lande et al., 2014; Zweifel‐Schielly et al., 2012).

As red deer populations in Switzerland (39,000+ individuals estimated in 2019) are on the rise, land use conflicts (e.g., impacts of ungulate herbivory on forest vegetation and agricultural production) will probably increase as well. In such cases, it is crucial to incorporate knowledge of space use patterns and foraging selectivity when searching for effective solutions to these conflicts. In this study, we linked migratory patterns of 93 collared red deer to their resource selection in open habitats and tested the GWH in four study areas in Switzerland. As recent advances in satellite remote sensing technology enable small‐scale landscape heterogeneity to be examined, we made use of Sentinel 2 satellite images to derive plant phenology and spatiotemporal forage availability. We tested four predictions within the framework of the GWH with special regard to its significance for red deer in human‐dominated landscapes. First, we expected red deer to select areas in open habitat at a state of green‐up (P1). Second, we predicted spring to arrive earlier in resident than in migrant summer ranges (P2), as we expected to see a delay of spring arrival in the summer home ranges of migratory individuals compared to winter home ranges, and hence predicted migratory individuals to have access to higher quality forage during spring green‐up than resident conspecifics (P3). Finally, we predicted green wave surfing performance of migrant red deer in Switzerland to be negatively influenced by the human‐altered landscape (P4).

## 2 MATERIAL AND METHODS

### 2.1 Study areas

**FIGURE 2:**
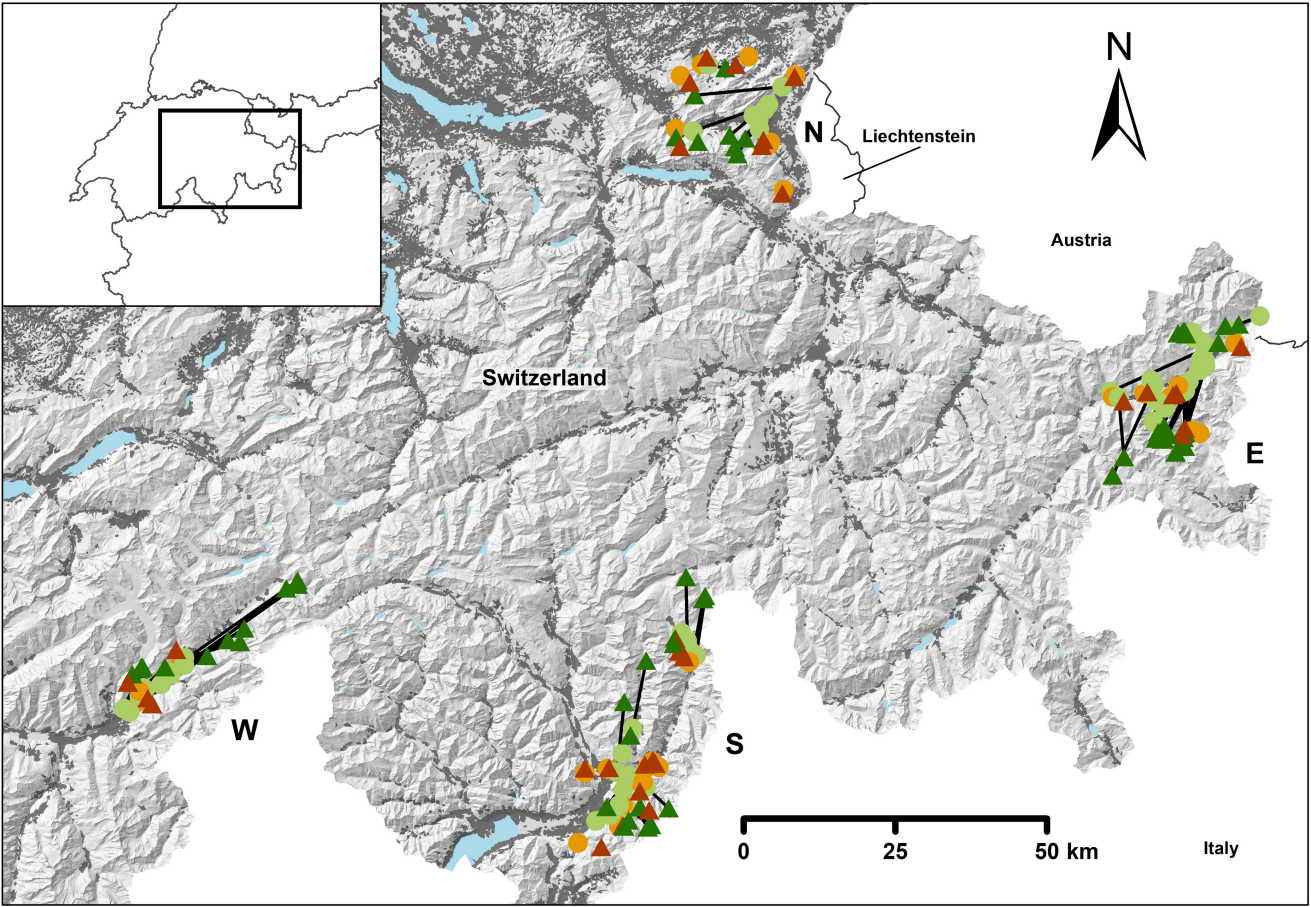
Overview of the study areas (capital letter). The background relief represents the topography of the Alps, the dark gray polygons are settlements, and the blue polygons are lakes. The light green circles denote the winter home ranges and the dark green triangles the summer home ranges (connected by lines) of migratory red deer. Winter and summer home ranges of resident individuals are indicated with orange circles and red triangles, respectively

We analyzed data from four study areas (Figure 2) that cover a substantial range of red deer habitats in the Alps: the northern Pre‐Alps and Alps (study area N, cantons of St. Gallen and Appenzell), inner alpine valleys of the east (study area E, Swiss National Park and surrounding areas in the canton of Grisons), the west (study area W, canton of Valais), and the southern fringe of the Alps (study area S, cantons of Grisons and Ticino). The two inner alpine study areas cover montane to alpine habitats with dry climates (E: av. 775 mm p.a.; W: av. 1064 mm p.a.), long winters and elevations ranging from 1000 to 4000 m a.s.l. The study areas at the northern and southern fringes are situated in colline to subalpine habitats with considerable precipitation (N: av. 1707 mm p.a., S: av. 1461 mm p.a.), moderate winters and elevations ranging from 300 to 2800 m a.s.l. The lower altitudes in all study areas are dominated by human activity (human footprint index (Wildlife Conservation Society, 2005): N = 17; E = 9.8; S = 12.8; W = 9.3) in the form of roads, settlements, agriculture, and forestry. Arable farming is mainly restricted to the bottoms of the main valleys in study areas N and S. Grassland and summer grazing by cattle and sheep are present in all study areas. Hunting is conducted outside game reserves according to cantonal regulations (study area E encompasses the Swiss National Park, where hunting is banned) in all of the study areas. Red deer hunting is generally practiced for a period of 3 weeks in September, except for parts of study area N (August 15th to December 15th). Supplementary feeding of ungulates in wintertime is not practiced in Switzerland.

### 2.2 Red deer data

We used spatial relocation data for female (58) and male (35) red deer that were captured and marked in a period from 2015 to 2018 (Study area N (2015–2016): *n* = 16; E (2015–2018): *n* = 31; S (2015–2018): *n* = 30; W (2017–2018): *n* = 16). Individuals were darted and immobilized at night in order to collar them (GPS telemetry collars; Vectronic Aerospace GmbH). They were then monitored over a period of 1–2 years, except for early drop‐offs due to fatalities or technical issues. Capturing, marking, and collaring were performed in the winter home ranges by authorities and game wardens of the respective cantons and were in line with Swiss animal welfare laws and approved by the appropriate authorities (permissions SG13‐12, GR2014‐07F, GR2015‐09, VS07‐17).

The sampling interval of the GPS collars was scheduled at 1–3 h, depending on the study area (N, E & W: 1‐2 h; S: 3 h). To ensure adequate locational accuracy, we only maintained GPS‐3D fixes in the datasets (95.6% of all fixes), which has proven to be the best screening option for reducing location error (Lewis et al., 2007).

### 2.3 Migratory patterns

To classify red deer migratory patterns, we used the net square displacement approach (NSD) to objectively categorize migratory behavior (Bunnefeld et al., 2011) into migration, mixed migration, nomadism, dispersal, and residency. We used the “MigrateR” R‐package (Spitz et al., 2017) that improves the classification of resident individuals, an issue that has been criticized previously (Bischof et al., 2012; Mysterud et al., 2011) and used the relative NSD function (rNSD) that selects the starting date and location based on the lowest AIC of a number of NSD models. We only classified movement behavior of individuals with a sampling period >9 months, to avoid problems with fitting movement models to trajectories considerably shorter than a year (Spitz et al., 2017). Because diurnal patterns were not of interest, we thinned the trajectories by averaging fixes to one location per day (Gurarie et al., 2017).

Migratory behavior does not need to be the same each year (Peters et al., 2019), therefore we split trajectories over multiple years into study years, with a starting date of February 1st each year. Accordingly, we set the starting location to this date (except for 30 animals that were captured after February 1st), as animals were assumed to remain in their winter ranges at this time of the year. We considered additional spatial constraints to help discriminate between migrant strategies: Migrants needed to have a minimum occupancy time in the summer range of 60 days and needed to have a minimum migration distance of 3 km between ranges, based on distances reported in other studies of red deer in the Alps (Georgii & Schröder, 1983 (on average 2.3 km nearest distance between seasonal ranges); Zweifel‐Schielly et al., 2009 (>3 km)). Additionally, we investigated if altitudinal migration could be detected in resident individuals (Spitz et al., 2017) and reclassified those with an elevational shift of seasonal ranges >500 m as migrants. Individuals classified as mixed migratory were reclassified as migratory because we intended to compare migrating subpopulations with resident ones (Peters et al., 2019).

### 2.4 Green wave modeling from satellite NDVI

Recent studies investigating the link between ungulate migration and the GWH have used MODIS terra‐derived NDVI as a proxy for forage quality and spatiotemporal dynamics (Aikens et al., 2017; Bischof et al., 2012; Merkle et al., 2016; Mysterud et al., 2017; Rivrud et al., 2016; Rivrud et al., 2018). One key issue is the relatively low granularity of MODIS terra data (250 m resolution), which means that different habitat types may be represented within one pixel. To account for this, we modeled plant phenology using images acquired by Sentinel 2 satellites (10 m resolution, revisiting time 5 days). Sentinel 2 Top‐Of‐Atmosphere reflectance products (from May 2017 on Bottom‐Of‐Atmosphere) are provided as open data by the European Space Agency (ESA).

Using Sentinel's Application Platform, we performed atmospheric corrections on the Top‐Of‐Atmosphere products with the Sen2Cor processor to obtain Bottom‐Of‐Atmosphere quality images. Subsequently, a scene classification was used to mask cloud, snow, or defective pixels in the Bottom‐Of‐Atmosphere bands prior to modeling the NDVI data.

We followed the protocol established by Bischof et al. (2012) and used these NDVI raster layers applying the modelNDVI function from the “phenex” R‐package (Lange & Doktor, 2017) to fit a double logistic function to our NDVI profile (with a best slope index extraction correction to reduce noise in the upper envelope) to model NDVI time series on open habitats for each year in each study area. We floored the values to the winter baseline (0.025 quantile) and applied a moving median filter (search window = 3) according to Bischof et al. (2012). We calculated the instantaneous rate of green‐up (IRG) as the rate of increase in NDVI values (scaled 0–1) between two successive days.

### 2.5 Step database

To test whether red deer select vegetation with intermediate biomass during spring green‐up, as defined by the IRG, we employed integrated step selection functions (iSSFs) using the “amt” R‐package (Signer, 2018). We first resampled animal trajectories at 3‐h intervals in order to regularize tracks over all of the study areas, and we only incorporated bursts with at least three consecutive steps in order to meet the minimum requirement for calculating turning angles. For each step, we then drew 25 random steps with potential target points by fitting a parametric distribution to the observed step lengths and turning angles, which resulted in a stratum of one used step versus 25 available steps (Merkle et al., 2016).

### 2.6 Explanatory variables

Using the step database, we extracted a set of explanatory variables at the used and available target points. We prepared a land use raster layer (categories: forest, barren land, unavailable (sealed land and waterbodies) and open habitat) based on official land surveys (swisstopo, 2018a). IRG and NDVI values were only assigned to target points on open habitats. Elevation and slope were extracted at the target points using the swissALTI3D digital elevation model (DEM) and slope dataset (swisstopo, 2018b), respectively. Based on the DEM we calculated solar radiation on a weekly basis using the area solar radiation tool in ArcGIS (version 10.5, ESRI). Using a canopy height model (Ginzler & Hobi, 2015), we calculated the vegetation cover of the shrub (0.5–3 m above ground) vegetation layer outside forested areas (e.g., hedges, shrubbery). All areal explanatory variables were rasterized at a 10 m resolution. To account for potential GPS location errors, we performed circular focal statistics (radius: 20 m, mean) on continuous variables (red deer GPS‐3D fixes reported with a mean location error of 13.9 m; Stache et al., 2012). Additionally, we measured Euclidean distances (all in m) from each target point to forest edges, and to roads and trails.

### 2.7 Spring green‐up in seasonal ranges

To investigate the arrival of spring green‐up in the red deer's winter ranges (mid‐Dec. to mid‐Mar.) and in the summer ranges (mid‐June to mid‐Sept.), we calculated the 95% utilization distribution (UD). This was performed by estimating the kernel density for the GPS data using the “adehabitatHR” R‐package (Calenge, 2006) with the ad hoc href smoothing factor. We only included individuals (*n* = 93) that had (i) data to calculate a UD for both seasons in the same year and (ii) at least 2 weeks of data (>112 fixes) for each season. We quantified the metrics of spring green‐up by taking the day of the year (DOY) of the start of the spring green‐up (threshold 0.01), of the peak spring green‐up (POS, threshold 0.5) and of the end of the spring green‐up (threshold 0.99) from the fitted NDVI curves (Lange & Doktor, 2017). By overlaying the green‐up metric raster with the UD we were able to establish the median day of the year (DOY) of the start of season, the median DOY of the peak spring green‐up and median DOY of the end of season for the corresponding ranges.

### 2.8 Statistical analysis

#### 2.8.1 Resource selection

To test for green‐up selection (P1) by red deer at the population level, we performed an integrated step selection analysis (iSSA (Avgar et al., 2017)) on the step database. We used a generalized linear mixed effects model with the step target points as the response variable and a Poisson distribution using the “glmmTMB” R‐package (Brooks et al., 2017). Following Muff et al. (2020), we used the stratum from the step database as a random intercept and fixed the variance at 10^6^ to avoid shrinkage. To ensure the movements of the deer corresponded to the green wave throughout the spring we reduced the step dataset for each red deer to the phase from the start of the spring green‐up in the winter range to the end of the spring green‐up in the summer range. This seems reasonable to ensure that some of the used and available target points correspond to spring green‐up (Merkle et al., 2016). Habitat selection by red deer can differ between day and night, due to differences in predation risk and human activity (Coppes et al., 2017; Godvik et al., 2009). However, results from a preliminary iSSA indicated no differences in open habitat selection between day and night (sun angle > 0 = day, night otherwise). We, therefore, used the pooled database to examine the factors that influence red deer open habitat selection. A preliminary iSSA showed an equal selection of open and forested habitats. Because NDVI cannot reflect forage availability in closed forest habitats (Borowik et al., 2013; Hamel et al., 2009) and because we were particularly interested in habitat selection mechanisms in open habitats, we restricted our analyses to open habitats. Also, pellet studies on the diet composition of red deer in Switzerland have shown that approximately 59% of the remnants of spring and summer diet belong to grassland species (Suter et al., 2004). Thus, we reduced the step dataset to open habitat target points and only kept records of the used step target point and at least three random target point per stratum. We screened covariates for collinearity using the Pearson correlation coefficient (|*r*_*p*_| < 0.7) and then parameterized a model that included IRG, NDVI, elevation, slope, solar radiation, shrub cover index, and minimum distance to forest edges, and to roads and trails. We included interactions between migrant strategies and the covariates to identify differences among residents and migrants. We also included step length as a covariate, since this can reduce potential sampling bias (Forester et al., 2009). Additionally, we included a random slope for all covariates (Muff et al., 2020), so that individuals nested in years and study areas could vary. Continuous covariates were rescaled by centering on the mean and dividing by the standard deviation, to avoid model convergence issues.

#### 2.8.2 Spring arrival in summer home ranges

Following Bischof et al. (2012), we calculated the difference in days between peak of spring green‐up in the summer and winter home ranges to test whether spring green‐up was delayed in the summer ranges of migratory individuals (P2). We fitted a linear mixed effects model with difference in days as the response variable, migrant strategy, sex, and study area as the fixed effects and the individual ID as the random intercept.

#### 2.8.3 Benefits of migration in plant green‐up

To assess whether migratory individuals have access to higher quality forage than their resident conspecifics (P3), we calculated the cumulative instantaneous rate of green‐up (CIRG) for each animal per study year by summing the IRG values of all used open habitat target points in the green‐up period, before fitting a linear mixed effects model with CIRG as the response variable. We used the estimated age of the individual (as estimated by experienced game wardens during capture), migrant strategy, and sex as parameters in the model and took the individual ID as random intercept.

#### 2.8.4 Green wave surfing

We tested green wave surfing in migratory individuals by applying a linear regression to estimate the relationship between date of peak green‐up and date of deer occupation on the open habitat target points (P4). We limited this part of the analysis to the spring green‐up phase, which we defined separately for each individual as the period between the start of season in the winter home range and the end of season in the summer home range. Based on the approach proposed by Aikens et al. (2017), we categorized the migrating deer into (i) theoretically perfect surfers; (ii) surfing performance better than random; or (iii) not surfing (cf. Aikens et al., 2017).

The absolute number of days from peak IRG (DFP) is the difference in days between the occupation date and the date of peak IRG. This index shows how well an individual surfs the green wave and is thus a measure of behavior. We used average DFP as the response variable in the general linear mixed effects model, mean distance to settlements and mean distance to roads as fixed effects, and the individual ID as random effect.

All statistical analyses and modeling steps were conducted using R version 3.5.1 (R Core Team, 2018).

## 3 RESULTS

### 3.1 Red deer migration patterns

Sixty‐nine point nine % of the monitored individuals (*n* = 65) showed migratory or mixed migratory behavior between their seasonal ranges. In females, 74.1% (*n* = 43) were considered migratory, and in males 62.9% (*n* = 22). All the other individuals (*n* = 28) were residents. The proportions of migratory individuals varied between study areas (N = 50%; S = 60%; E = 77%; W = 94%; Table A1). The average distance traveled by migratory red deer was 12.1 km (SD: 8.86 km, females: 11.4 km, males: 13.5 km). The average distances migrated varied between study areas from 7.66 km (SD: 4.58 km, N) to 15.6 km (SD:10.6 km, W). Over all study areas, migrants remained 116 days (SD: 50 days, female: 121 days, male: 106 days) in their summer home ranges. The duration of stay varied between study areas from 100 days (SD: 35 days, E) to 136 days (SD: 75 days, N). On average migration started on 30. April (SD: 33 days, female: 23. April, male: 16. May) at the winter range (Table A2).

### 3.2 Resource selection

**FIGURE 3:**
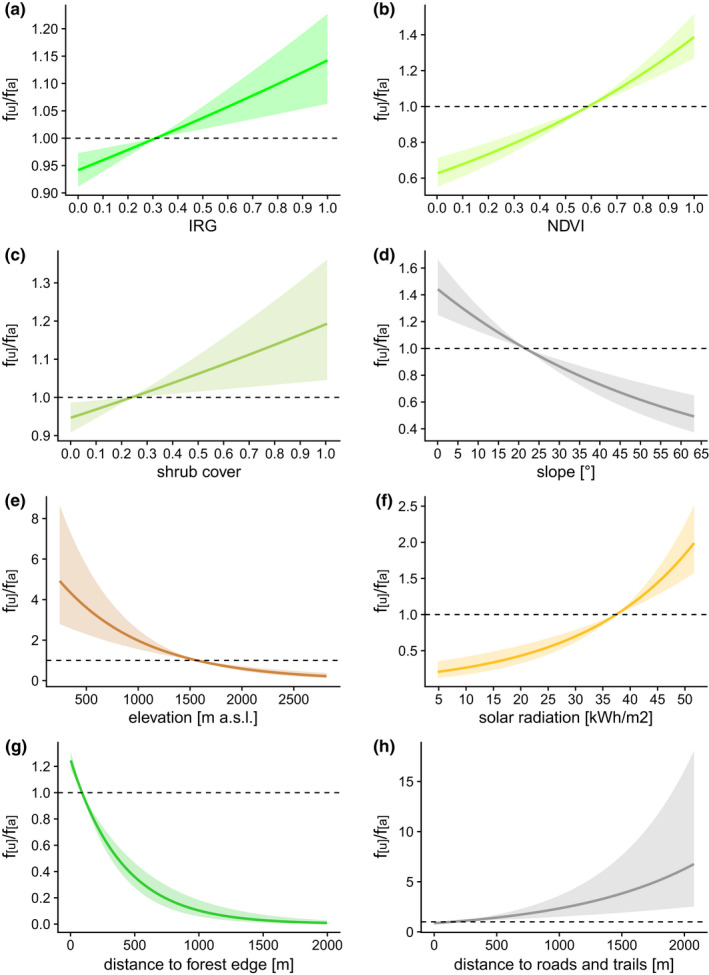
Relationship between selection and IRG (a), NDVI (b), shrub cover (c), solar radiation (d), slope (e), elevation (f), distance to forest edge (g), and distance to roads (h) for migrants. Values >1 indicate preference, whereas values <1 indicate avoidance. The proportion *f*_*u*_/*f*_*a*_ relates to used and available frequencies. Shaded areas encompass all pointwise 95% confidence intervals. Probability of selection (*f*_*u*_/*f*_*a*_) is based on predicted values of a step selection analysis parametrized with GPS collar data

In line with our first prediction (P1), migratory (*β* = 0.05, SE = 0.02, *p* = .001) and resident (*β* = −0.00, SE = 0.03, *p* = .923) red deer selected spring green‐up in open habitats (Table 1; Figure 3a). Furthermore, they selected areas with higher NDVI values (migrants: *β* = 0.19, SE = 0.03, *p* < .001) and habitat with shrub cover (migrants: *β* = 0.06, SE = 0.02, *p* = .016; Figure 3b,c). They also selected cells with lower elevation (migrants: *β* = −0.47, SE = 0.11, *p* < .001; Figure 3e). Migratory red deer selected habitat patches closer to forest edges (*β* = −0.33, SE = 0.04, *p* < .001) compared to residents (*β* = 0.19, SE = 0.09, *p* = .035), but all individuals selected zones further away from roads and trails (migrants: *β* = 0.15, SE = 0.06, *p* = .008; Figure 3g,h). Migratory individuals showed a strong selection for cells with higher solar radiation values (*β* = 0.38, SE = 0.06, *p* < .001; Figure 3f), whereas resident individuals preferably used cells with lower solar radiation values (*β* = −0.33, SE = 0.04, *p* = .003). They also selected less sloping terrain (*β* = −0.12, SE = 0.04, *p* = .002) and residents selected even less steep terrain (*β* = −0.20, SE = 0.08, *p* = .011).

**TABLE 1:** Red deer habitat selection estimates during the green‐up season. A step selection analysis was parametrized by data from 93 individuals with a generalized linear mixed effects model. The model estimate, its standard error, its corresponding confidence interval (CI), and the associated *p*‐value (*p*) are listed for each model covariate

Predictors	*β*	SE	CI	*p*
(Intercept)	−24.13	2.03	−28.10 to −20.16	**<.001**
IRG	0.05	0.02	0.02–0.09	**.001**
NDVI	0.19	0.03	0.13–0.26	**<.001**
Elevation	−0.47	0.11	−0.69 to −0.26	**<.001**
Slope	−0.12	0.04	−0.20 to −0.04	**.002**
Solar radiation	0.38	0.06	0.27–0.49	**<.001**
Shrub cover index	0.06	0.02	0.01–0.10	**.016**
Distance to forest edge	−0.33	0.04	−0.41 to −0.25	**<.001**
Distance to roads and trails	0.15	0.06	0.04–0.26	**.008**
Step length	1.17	0.09	0.99–1.35	**<.001**
IRG:residents^a^	−0.00	0.03	−0.07 to 0.06	.923
NDVI:residents^a^	0.05	0.07	−0.08–0.19	.422
Elevation:residents^a^	−0.34	0.24	−0.80–0.13	.153
Slope:residents^a^	−0.20	0.08	−0.35 to −0.05	**.011**
Solar radiation:residents^a^	−0.33	0.11	−0.54 to −0.11	**.003**
Shrub cover index:residents^a^	−0.01	0.05	−0.10–0.08	.812
Distance to forest edge:residents^a^	0.19	0.09	0.01–0.37	**.035**
Distance to roads and trails:residents^a^	0.24	0.13	−0.01–0.49	.065
N stratum	12,009			
N id	83			
N year	3			
Random effects	*σ* ^2^			
IRG	0.0368			
NDVI	0.2135			
Elevation	0.6169			
Slope	0.2531			
Solar radiation	0.3354			
Shrub cover index	0.1236			
Distance to forest edge	0.2253			
Distance to roads and trails	0.3209			
Step length	0.7355			

*Notes*: Bold indicates statistically significant *p*‐value.

^a^ Migrants.

### 3.3 Spring arrival in summer home ranges

**FIGURE 4:**
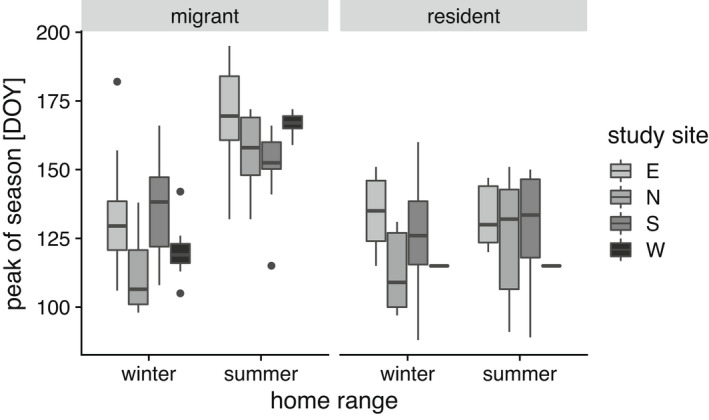
Box‐whisker‐plot of median day of the year (DOY) of peak spring green‐up (peak of season POS) in the corresponding range. Median POS shown separately for winter and summer home ranges of migratory and resident individuals in each study area

Consistent with P2, peak of spring green‐up (POS) occurred on average 36 days (SD: 21 days) later in the summer home ranges of migratory individuals than in their winter range (Figure 4), whereas delay of POS at summer ranges of resident animals (on average 6 days, SD: 11 days) was significantly shorter (*β* = −29.02, SE = 3.9, *p* < .001, Table 2). For 62 migrant red deer (95.3% of all migrating individuals), the POS occurred later in the summer range than in the winter range.

**TABLE 2:** Model predicting difference in peak of spring green‐up (POS) arrival between winter and summer ranges in resident and migratory red deer. POS as a function of migration tactic, sex, and study area. The model estimate, its standard error, its corresponding confidence interval (CI), and the associated *p*‐value (*p*) are listed for each model covariate

Predictors	*β*	SE	CI	*p*
(Intercept)	35.38	4.00	27.53–43.23	**<.001**
Residents^a^	−29.02	3.90	−36.66 to −21.37	**<.001**
Male^b^	2.75	3.59	−4.29–9.79	.444
Study area N^c^	4.57	5.45	−6.11–15.24	.402
Study area S^c^	−13.91	4.44	−22.62 to −5.20	**.002**
Study area W^c^	11.11	5.52	0.28–21.93	**.044**
Random effects				
*σ* ^2^	124.83			

*Notes*: Bold indicates statistically significant *p*‐value.

^a^ Migrants.

^b^ Female.

^c^ Study area E.

### 3.4 Benefits of migration regarding plant green‐up

Migratory individuals had access to significantly higher CIRG (*β* = 11.29, SE = 5.48, *p* = .039) than their resident conspecifics. No differences could be found between the sexes, nor had the estimated age of the study animals a significant effect on their access to high‐quality forage (Table 3).

**TABLE 3:** Model predicting access to high‐quality forage in resident and migratory red deer during spring green‐up. Cumulative instantaneous rate of green‐up (CIRG) as a function of migration tactic, sex, and age. The model estimate, its standard error, its corresponding confidence interval (CI), and the associated *p*‐value (*p*) are listed for each model covariate

Predictors	*β*	SE	CI	*p*
(Intercept)	34.34	7.57	19.50–49.19	**<.001**
Migrants^a^	11.29	5.48	0.56–22.03	**.039**
Male^b^	−3.98	5.37	−14.50–6.54	.458
Age	0.52	0.75	−0.94–1.99	.484
Random effects				
*σ* ^2^	317.68			

*Notes*: Bold indicates statistically significant *p*‐value.

^a^ Residents.

^b^ Female.

### 3.5 Green wave surfing

Only 14/62 (22.6%) migratory red deer with a peak of spring green‐up that occurred later in their summer ranges than in their winter ranges proved to be perfect surfers (Table A3). Even so, 47/62 (75.8%) individuals surfed the green wave better than at random, whereas 15/62 (24.2%) individuals did not surf at all. In the less populous alpine study areas (human footprint index: E = 9.8/W = 9.3), higher proportions of the migrating subpopulation (E = 79%/W = 100%) surfed the green wave than in the more populous areas (human footprint index: N = 17/S = 12.8) at the fringes of the Alps (N = 50%/S = 60%; Table A3).

Red deer surfed the green wave closer to peak green‐up when more distant to settlements (*β* = −0.18, SE = 0.04, *p* < .001), while the distance to roads did not have an influence on days from peak IRG (Table 4).

**TABLE 4:** Model predicting days‐from‐peak green‐wave surfing as a function of distance to settlement and distance to roads. The model estimate, its standard error, its corresponding confidence interval (CI), and the associated *p*‐value (*p*) are listed for each model covariate

Predictors	*β*	SE	CI	*p*
(Intercept)	2.34	0.05	2.25–2.43	**<.001**
Distance to settlements	−0.18	0.04	−0.26 to −0.10	**<.001**
Distance to roads	0.05	0.04	−0.03–0.13	.247
Random effects				
*σ* ^2^	0.61			

*Note*: Bold indicates statistically significant *p*‐value.

## 4 DISCUSSION

In this study, we evaluated the influence of spatiotemporal variability in forage quality and a set of environmental variables on habitat selection of red deer and their migrant strategies during the spring green‐up phase in a human‐dominated landscape. Habitat selection of red deer was influenced by the stage of green‐up and therefore forage quality (P1). However, it depended also on the migrant strategy, landscape characteristics and proxies for human presence. In the summer home ranges of migrants, green‐up peaked later than in the summer home ranges of residents (P2). During the green‐up phase, migratory individuals had access to higher quality forage than resident conspecifics (P3). 76% of the red deer exhibited green wave surfing behavior that was better than at random, and surfing performance was negatively influenced by the proximity of settlements (P4).

### 4.1 Resource selection: Green‐up versus landscape structure and human presence

After winter, when deer body fat reserves are reduced (Arnold, 2020), the availability of emerging spring green‐up can be a strong driver for ungulate habitat selection in seasonal landscapes (Laforge et al., 2021). We found evidence that red deer select for zones with emerging vegetation, an indication of high‐quality forage, as the collared individuals selected locations with a higher IRG compared to the average available at random locations. Furthermore, the positive selection for IRG values indicates that red deer follow the green wave, which agrees with current research (Merkle et al., 2016). Additionally, we found a strong selection for higher NDVI values in comparison to the average available at random locations in open habitats. This result may be related to red deer selecting for high plant biomass toward the end of the green‐up phase in their summer home range when the green wave is mostly over. Selection for areas with shrub cover indicates that shrub biomass may be important as food resource (Hebblewhite et al., 2008) or cover.

Our findings show a selection of open habitat further away from roads and trails, which is presumably a reaction to disturbances caused by human activities and traffic (cf. Coppes et al., 2017; Roberts et al., 2017). Forage on patches near roads and trails in the study areas is undoubtedly just as palatable as anywhere else, hence avoiding these areas may be fitness relevant as the available foraging area is reduced. Grazing in such areas causes a trade‐off, since vigilance behavior would likely increase (Ciuti et al., 2012) and energetically costly flights become more probable (Wisdom et al., 2018). As a result, red deer may concentrate in areas with less human activities, where an increase in the use of forage could amplify land‐use conflicts.

We found migratory red deer avoid areas far away from forest edges when in open habitats, which may arise from an increased need for safety. For example, elk shifted their habitat selection of open areas more toward forest edges after the reintroduction of the wolf in Yellowstone (Hernández & Laundré, 2005). Moreover, the shift toward forest edges led to poorer quality nutrition for these elk compared to individuals in wolf‐free reference areas. Because wolf numbers in Switzerland were low at the time and just very few of the collared red deer may have experienced wolf contact throughout their lives, we argue that this behavior was a consequence of human activity and in particular non‐hunting activities, since the hunting season does not start until 15th of August at the earliest.

In general, topography and landscape structure restrict space use by red deer. The avoidance of high altitudes and steep slopes is related to the high, barren mountain tops and the rugged alpine landscape, suggesting that less steep habitat patches provide higher forage availability. Patches with high solar radiation values are generally southerly exposed and with the onset of spring are among the first to become free of snow and to turn green and are thus particularly preferred during this phase.

### 4.2 Benefits of migration regarding plant green‐up

A considerable proportion of the study animals were classified as migratory, though this proportion varied between study areas with an increased propensity for red deer migration in inner alpine habitats with strong seasonal variability (cf. Peters et al., 2019). Our findings indicate that migratory behavior results in better access to high‐quality forage than resident behavior, which is in line with current research on the benefits of migration regarding plant green‐up (Bischof et al., 2012; Hebblewhite et al., 2008; Mysterud et al., 2017).

Other environmental or intrinsic factors certainly also play a role in favoring the decision to migrate (e.g., higher probability to migrate as densities increase; Mysterud et al., 2011). Predation can pose a risk to migrating animals, since exposure to predation risk has been shown to be 1.7 times higher during migration than for resident individuals (Hebblewhite & Merrill, 2007). Indeed, brown bear (*Ursus arctos*) density had a negative influence on CIRG for migrating semi‐domestic reindeer (*Rangifer tarandus*) (Rivrud et al., 2018). However, as wolf abundance was low in our study areas, this was probably a minor threat to migrating red deer.

### 4.3 Green wave surfing

We predicted that green wave surfing performance was negatively influenced by the human‐dominated landscape. Thus overall, did not expect to see 76% of the individuals to surf better than at random. Nevertheless, it has been shown that in the more sparsely populated study areas, the proportion of surfing individuals is higher than in the more densely populated areas. Also, green wave surfing performance was better in migrating individuals that used zones more distant from settlements than the ones that stayed closer to villages. Human activities and disturbance near settlements probably cause a restriction of mobility (motorways, fences, urban areas) and access to forage grounds close to peak green‐up. However, other reasons may also explain why some individuals (24%) deviate from surfing the green wave, such as spatio‐temporal variation in green‐up (Martin et al., 2018), density dependence or jumping the green wave in order to arrive in the summer ranges to fully exploit spring green‐up there (Bischof et al., 2012; Laforge et al., 2021).

Empirical evidence has been found that surfing of the green wave by elk can be fitness relevant, and simulations have shown pregnancy rates and population size decrease as the mismatch between the date of patch occupancy and the date of peak green‐up increases (Middleton et al., 2018). In our system however, these links need further investigation, since the influence of intensive grassland use and the climate change‐driven alteration of the phenology of key food resources on red deer migration are currently unexplored. Aikens et al. (2020) have shown that a shorter window of green‐up caused by drought events reduced the opportunity to accumulate forage resources during spring migrations. We argue that intensified use of meadows in terms of fertilization and frequent mowing increases the proportion of open areas providing high‐quality forage (c.f. Smit et al., 2008). Combined with climate warming effects, this development probably prolongs the time window of grassland food supply in Central European habitats. Thereby, current land use and climate warming further red deer population growth and thus potentially amplify conflicts red deer cause with forestry production and agriculture.

### 4.4 Conclusions and management implications

Our work demonstrates the influence of human activities and man‐made habitat alterations on resource selection by red deer. Selection of high‐quality forage has proven to be restricted by human presence; therefore, it is important to provide disturbance‐free areas in the sensitive period in early spring. Nevertheless, within the limits of topographical constraints, red deer track green‐up by selecting cells with high IRG values on a fine spatiotemporal scale and by following the green wave across landscapes in migratory movements. Thus, our analysis also supports the key assumptions of the GWH in human‐dominated landscapes with small‐scale heterogeneity and vegetation emerging in a heterogenic, dynamic mosaic. Consequently, red deer are obviously capable of benefitting from the patchy but high forage supply in intensely used landscapes. Our results can help managers to improve spatially explicit planning in ungulate management systems to reduce feeding pressure on cultivated open land or forests and thereby reduce conflicts.

## AUTHOR CONTRIBUTIONS

**Benjamin Sigrist:** Conceptualization (equal); data curation (supporting); formal analysis (lead); investigation (supporting); project administration (lead); visualization (lead); writing – original draft (lead); writing – review and editing (equal). **Claudio Signer:** Data curation (equal); funding acquisition (equal); project administration (supporting); writing – original draft (supporting). **Sascha Wellig:** Data curation (equal); investigation (equal); writing – original draft (supporting). **Arpat Ozgul:** Conceptualization (supporting); supervision (supporting); writing – original draft (supporting). **Flurin Filli:** Data curation (equal); investigation (equal); writing – original draft (supporting). **Hannes Jenny:** Data curation (equal); investigation (equal); writing – original draft (supporting). **Dominik Thiel:** Data curation (equal); investigation (equal); writing – original draft (supporting). **Sven Wirthner:** Data curation (equal); investigation (equal); writing – original draft (supporting). **Roland Graf:** Conceptualization (supporting); formal analysis (supporting); funding acquisition (equal); project administration (supporting); supervision (lead); writing – original draft (supporting); writing – review and editing (equal).

## CONFLICT OF INTEREST

We declare no conflict of interest.

## Data Availability

Data are available on Dryad Digital Repository (https://doi.org/10.5061/dryad.1vhhmgqw9).

## APPENDIX A

**TABLE A1:** Migratory and resident red deer listed respective to sex and study area

	Study area N	Study area E	Study area S	Study area W
Migratory female	6	13	13	11
Migratory male	2	11	5	4
Resident female	4	6	5	‐
Resident male	4	1	7	1

**TABLE A2:** Migration parameters of migrating individuals, listed as entire migrating sub‐population (all), separated into sex (m/f), and study area (E/N/S/W). Parameters include average (Av) and standard deviation (SD) of migrated distance, Av and SD days spent at summer home range (shr), Av and SD days spent migrating, Av and SD day of the year (DOY) migration started at the winter range

	Av dist	SD dist	Av days shr	SD days shr	Av mig (days)	SD mig (days)	Av. mig DOY	SD Mig DOY
All	12.1	8.86	116	50	8.26	8.05	120	33
f	11.4	8.68	121	53	8.98	8.24	113	32
m	13.5	9.29	106	43	6.79	7.66	136	32
E	11.1	8.53	100	35	5.33	7.59	146	35
N	7.66	4.58	136	75	13.1	8.37	80	16
S	12.3	8.45	126	63	8.89	7.79	107	28
W	15.6	10.6	119	42	9.2	7.93	118	17

**TABLE A3:** Surfing performance of 62 migratory red deer listed respective to study area

	Study area N	Study area E	Study area S	Study area W
Perfect surfing	–	8	5	1
Better than random	4	11	4	14
No surfing	4	5	6	–
